# Knowledge, attitudes, and practices regarding tick-borne diseases among an at-risk population living in Niigata prefecture, Japan

**DOI:** 10.1371/journal.pone.0270411

**Published:** 2022-06-30

**Authors:** Taichi Narita, Hansani Madushika Abeywickrama, Marcello Otake Sato, Kozo Watanabe, Reiko Arai, Tsutomu Tamura, Megumi Sato

**Affiliations:** 1 Graduate School of Health Sciences, Niigata University, Chuo-ku, Niigata City, Japan; 2 Laboratory of Tropical Medicine and Parasitology, Dokkyo Medical University, Mibu-machi, Shimotsuga-gun, Tochigi, Japan; 3 Center for Marine Environmental Studies (CMES), Ehime University, Matsuyama, Ehime, Japan; 4 Niigata Prefectural Institute of Public Health and Environmental Sciences, Nishi-ku, Niigata City, Japan; Management and Science University, MALAYSIA

## Abstract

**Objective:**

This study examined the knowledge, attitudes, and practices (KAP) of an at-risk population living in Niigata prefecture regarding tick-borne diseases (TBDs) and preventive strategies.

**Methods:**

A cross-sectional questionnaire-based study was conducted to assess the KAP of the community.

**Results:**

In total, 186 responses were received. Among the respondents, 130 (69·9%) were men, and the mean age was 51.1 (14·3). Nine (4·8%) respondents reported having experienced tick bites. Of the respondents, 44 (23.7%) knew about both scrub typhus and severe fever with thrombocytopenia syndrome, while 156 (83·9%) and 71 (38·2%) recognized limiting skin exposure and use of insect repellents as preventive measures, respectively. The attitudes towards TBDs: being worried about tick bites (p = 0·018) and interested in preventing TBDs (p = 0·001), were significantly higher among women than men. About 75% of the respondents reported taking preventive measures against tick bites, and limiting skin exposure was the most frequently applied method (69·9%). Insect repellents were used by 58 (31·2%) respondents. Age (p = 0·049), being worried about tick bites (p = 0·046), and knowledge of ticks score (p = 0·024) were the significant independent predictors of practicing countermeasures.

**Conclusion:**

We identified gaps in knowledge and practices regarding TBDs. Public health interventions should be implemented to improve public awareness of TBDs.

## 1. Introduction

Ticks are hematophagous vectors that transmit various pathogens (viruses, bacteria, rickettsiae, spirochetes, and protozoa) [1] and cause tick-borne diseases (TBDs) such as tick-borne encephalitis (TBE), rocky mountain spotted fever, and Crimean Congo hemorrhagic fever [2]. Over the past few decades, there has been a rapid spread of TBDs, resulting in public health risk worldwide owing to population mobility, climate change, animal migration, and global logistics [2]. Today, ticks are widely distributed in human habitats, especially in areas with much vegetation [3]. The list of notifiable diseases (Infectious diseases categories I and IV) in Japan contains 12 TBDs, so reporting is mandatory when a patient is diagnosed with those diseases. The most commonly observed TBDs in Japan are Japanese spotted fever (JSF), Lyme borreliosis (LB), and scrub typhus. In addition, severe fever with thrombocytopenia syndrome (SFTS) and TBE have caused several fatal cases in Japan [2, 4].

The factors that determine the risk of becoming infected with a TBD are; (1) the overall number of ticks in the area (density of the tick population), (2) the proportion of ticks carrying a pathogen (infection rate), and (3) human behavior that influence the risk of exposure [5, 6]. People engaged in outdoor occupational or recreational activities (farming, military training, forestry, hunting, fishing, and camping) in risk areas are at high risk of being bitten by ticks [7, 8]. Individual protective measures are commonly recommended to prevent tick bites, such as avoid risk areas or stay on trails while in risk areas, minimize skin exposure (light-colored clothing, wear long sleeves and trousers, tucking trousers into socks, and wearing rubber boots), use of repellents, check the body if exposed to ticks, and remove ticks as soon as possible after they attach [5, 9, 10]. When removing an attached tick using a tweezer or a fine-pointed forcep, caution must be taken not to break off the mouthparts. Therefore, the general public should be informed about the correct method of removing ticks or doing it at a medical site [6]. The most common policy measure used by health authorities to reduce TBDs is to launch information campaigns and education programs aimed at avoiding tick bites [11].

Recently, we performed a tick survey in Niigata prefecture in Japan after 60 years [12], since the large-scale tick survey conducted by Saito in 1959 [13]. The survey was conducted in 41 sites in the prefecture where people are likely to be exposed to ticks, including parks, trails, forests, and camping areas. We found 12 species of ticks, and the three most common species in the area were *Haemaphysalis flava*, *Haemaphysalis longicornis*, and *Ixodes ovatus*. These three tick species harbor the pathogens causing JSF, LB, and SFTS [12]. We also found three species of spotted fever group rickettsiae; R*ickettsia asiatica*, *Rickettsia helvetica*, and *Rickettsia monacensis*, in ticks in Niigata Prefecture, Japan [4]. As we found evidence on Niigata prefecture as a risk area to TBDs, it is essential to take measures to improve awareness for avoiding tick bites and disease prevention.

Knowledge, attitude, and perception (KAP) studies are used to investigate health behavior and are essential in developing effective public health strategies [14]. Despite the increasing incidences, very limited scientific evidence is available on KAP regarding TBDs in the Japanese population. In Japan, only one study conducted in Hokkaido has assessed the awareness of ground defense force officers regarding tick-borne infections [15]. Therefore, the present study aimed to assess KAP regarding TBDs among an at-risk population in Niigata prefecture in Japan, focusing on the respective preventive measures.

## 2. Materials and methods

### 2.1. Study design and study setting

A cross-sectional survey was conducted from February 2019 to March 2020 in Niigata prefecture, Japan. Niigata prefecture stretches along the coast of the sea of Japan from the southwest to the northeast. It has an area of 12,584 square km, with a population of about 2.17 million (as of December 2021) [16]. It is a major agricultural prefecture in Japan, with many mountainous areas. Niigata’s vast forests cover up more than 60% of the land. As mentioned earlier, recent studies have identified Niigata prefecture as a high-risk area for TBDs [4, 12].

### 2.2. Study sample and procedures

The sample size was calculated by multiplying the number of variables used in the multiple regression model by 10, which resulted in 170 (17 x 10). We decided to distribute a total of 340 questionnaires, assuming a response rate of 50%.

The study population was people in Niigata prefecture who are at risk of contracting TBDs.

The sampling method was based on time-method sampling (TLS), a probabilistic sampling method used to recruit members of a target population. In TLS, the assumption is that it is possible to access the target population in predefined venues. Therefore, People who visited the agriculture departments in prefecture branch offices were targeted for this study, as they were involved in agriculture and forestry and at risk of contracting TBDs. People who visited the agriculture departments were introduced to the research, and the questionnaire folder was handed over to those interested in participating in the survey. Those who were non-residents in the Niigata prefecture and <18 years old were excluded. The participants were invited to participate in the study in writing through an information sheet provided with the questionnaire.

### 2.3. Study instrument

A self-administered questionnaire was constructed to collect information and consisted of a total of 17 items in 5 domains. Five items assessed the basic characteristics of respondents, including three items on demographic characteristics (gender, age, and the type of household), the experience of being bitten by ticks (1 item: yes/ no question), and the information sources used to get information on prevention of tick bites (1 item: maximum of 3 answers were allowed). Knowledge of ticks (1 item: knowledge of ticks score), TBDs (1 item: multiple answers were allowed), and preventive measures (1 item: multiple answers were allowed) were assessed by three items. Knowledge of ticks score was calculated by using four statements with yes/ no options; (i) There is no risk of tick bites in daily life., (ii) Ticks are distributed only in the western part of Japan., (iii) There are no health risks associated with ticks., and (iv) There is no need to go to a hospital if you get bitten by a tick. For all the statements, 1 point was given if the answer was ‘no’ and 0 points to the answer ‘yes’; hence the knowledge of ticks score ranges from 0–4.

The attitude domain was assessed by two items on being worried about tick bites (1 item: yes/ no question) and interested in taking preventive measures (1 item: yes/ no question). The practice domain was assessed by the practice of preventive measures against ticks (1 item: yes/no question) and preventive measures that were practiced (1 item: multiple answers were allowed). The health literacy (HL) was assessed using a scale developed by Ishikawa *et al*. (2008) [17], which contained three items for communicative HL and two items for critical HL. The items that assessed the HL were the ability to; (i) collect health-related information from various sources, (ii) extract the required information, (iii) understand and communicate the obtained information, (iv) consider the credibility of the information, and (v) make decisions based on the information. Each item was rated on a 5-point scale, ranging from 1 (‘strongly disagree’) to 5 (‘strongly agree’). The questionnaire was prepared and administered in the Japanese language.

### 2.4. Ethical considerations

Ethical clearance was obtained from the Ethical Review Committee on research involving human subjects in Niigata University, Japan (No. 2017–0140). Each participant received an explanation about the study and the opt-out to respond/ return the questionnaire. The return of the completed questionnaire was considered as the consent.

### 2.5. Statistical analysis

The analysis of descriptive statistics was conducted by gender and age. Descriptives were presented as frequencies (n) and proportions (%) for categorical variables and the mean values and standard deviations (SD) for continuous variables. As we excluded the missing values, the number of total responses was not always the same. Therefore, the proportions were calculated using the total number of responses for each item as the denominator. Significant differences in proportions between groups were tested by the chi-square test or Fisher’s exact probability tests. For the continuous variables, the t-test for independent samples was used to compare the two groups. Differences were regarded as significant at a two-tailed *p*-value <0.05. Finally, a multiple logistic regression analysis was performed to identify the factors associated with KAP, using the practice of countermeasures against ticks as the dependent variable. Independent variables with a *p*-value <0.2 in the univariate analysis (age, sex, type of household, sources of information, knowledge of ticks score, HL score, worried about being bitten by ticks, and interested in taking countermeasures) were included in the multiple regression model. All analyses were conducted using SPSS 27.0 (IBM Inc., Tokyo, Japan).

## 3. Results

Out of 340 questionnaires distributed, 186 completed questionnaires were received (response rate: 51·7%). Among the respondents, 130 (69·9%) were men, while the mean (SD) age was 51·1 (14·3). More than half of the respondents (n = 103, 56·3%) had nuclear families, while 62 (33·9%) had three-generation families. Approximately 7% (n = 9) reported having experienced tick bites, and among them, 8 (88·9%) were men, and 6 (66·7%) were <65 years old. Internet was the leading source of information for most respondents (n = 80, 43·0%) which followed by television (n = 64, 34·4%) and newspapers (n = 50, 26·9%). The use of official publications as the source of information was significantly higher among women than men (*p* = 0·020), while the use of the internet for information was significantly higher among <65 years old respondents (*p* = 0·010) (Table 1).

**Table 1:** Basic characteristics of respondents with comparison by age and sex.

	Total	By sex			By Age		
		Male	Female	*p*	< 65 years	≥ 65 years	*p*
Age ^a^; Mean (SD)	51·08 (14·33)	50·93 (14·37)	51·44 (14·37)	0·827			
Sex ^b^							
Male	130 (69·89)				102 (70·35)	26 (68·42)	0·844
Female	56 (30·11)				43 (29·65)	12 (31·58)	
Type of household ^b^							
Living alone	15 (8·20)	8 (6·25)	7 (12·73)	0·216	13 (9·09)	1 (2·63)	0·005*
Nuclear family	103 (56·28)	70 (54·69)	33 (60·0)		71 (49·65)	31 (81·58)	
Three-generation	62 (33·88)	47 (36·72)	15 (27·27)		56 (39·16)	6 (15·79)	
Other	3 (1·64)	3 (2·34)	0 (0·0)		3 (2·10)	0 (0·0)	
Experience of being bitten by ticks ^c^							
Yes	9 (6·82)	8 (8·79)	1 (2·44)	0·273	6 (5·77)	3 (11·54)	0·382
No	123 (93·18)	83 (91·21)	40 (97·56)		98 (94·23)	23 (88·46)	
Sources of Information ^b^^#^							
Newspaper	50 (26·88)	30 (23·08)	20 (35·71)	0·104	35 (24·14)	14 (36·84)	0·149
Magazines	20 (10·75)	11 (8·46)	9 (16·07)	0·130	17 (11·72)	3 (7·90)	0·770
Books	18 (9·68)	10 (7·69)	8 (14·29)	0·182	15 (10·35)	3 (7·90)	0·769
TV	64 (34·41)	43 (33·08)	21 (37·50)	0·615	47 (32·41)	15 (39·47)	0·444
Radio	30 (16·13)	22 (16·92)	8(14·29)	0·828	24 (16·55)	5 (13·16)	0·804
Internet	80 (43·01)	55 (42·31)	25 (44·64)	0·872	68 (46·90)	9 (23·68)	0·010*
Official publications	33 (17·74)	17 (13·08)	16 (28·57)	0·020*	26 (17·93)	7 (18·42)	1·000

Data are presented as numbers and proportions (%) unless otherwise specified.

Missing values were excluded, and the proportions were calculated using the total number of responses for the respective section as to the denominator.

*Significant differences between groups at *p* <0·05.

^#^Maximum 3 answers were allowed.

^a^ Student’s t-test

^b^ Chi-square test

^c^ Fisher’s exact test

### 3.1. Knowledge

The mean (SD) knowledge of ticks score was 2·8 (1·3), with no significant differences in the score with sex and age categories (Table 2). Among the respondents, 44 (23·7%) knew about scrub typhus and SFTS, and 24 (12·9%) were aware of JSF. Moreover, the knowledge about JSF (*p* = 0·001), LB (*p* <0·001), and tularemia (*p* = 0·006) were significantly higher among women than men. No significant differences in knowledge about TBDs were found between the two age groups. Regarding knowledge about preventive measures, the majority (83·9%) of respondents answered limiting skin exposure. Only 71 (38·2%) of respondents identified insect repellents as a preventive measure, while the knowledge of repellents was significantly higher among women (*p* = 0·001). The preventive measures mentioned under the ‘other’ option were ‘not going out’, ‘wear boots, rain gear, and long pants’, ‘not wearing white clothes’, and ‘sprinkling salt over clothes’. Four respondents mentioned that they do not know about the preventive measures. The mean (SD) communicative, critical, and total HL scores were 10·5 (2·3), 6·5 (1·5) and, 16·9 (3·5), respectively.

**Table 2:** Knowledge of TBDs and health literacy by sex and age.

	Total	By sex			By age		
		Male	Female	*p*	<65 years	≥65 years	*p*
Knowledge of ticks score; mean (SD) ^a^	2·8 (1·3)	2·7 (1·4)	2·9 (1·2)	0·274	2·9 (1·2)	2·4 (1·5)	0·051
Knowledge of TBDs ^**b**^^**#**^							
Scrub typhus	44 (23·7)	27 (20·8)	17 (30·4)	0·189	31 (21·4)	13 (34·2)	0·134
JSF	24 (12·9)	9 (6·9)	15 (26·8)	0·001*	20 (13·8)	4 (10·5)	0·789
LB	14 (7·5)	3 (2·3)	11 (19·6)	<0·001*	12 (8·3)	2 (5·3)	0·738
Tularemia	14 (7·5)	5 (3·8)	9 (16·1)	0·006*	11 (7·6)	3 (7·9)	1·000
SFTS	44 (23·7)	27 (20·8)	17 (30·4)	0·189	37 (25·5)	7 (18·4)	0·403
Knowledge of preventive measures ^**b**^^**#**^							
Limiting skin exposure	156 (83·9)	108 (83·1)	48 (85·7)	0·828	121 (83·4)	33 (86·8)	0·804
Wear light-colored clothes	5 (2·7)	2 (1·5)	3 (5·4)	0·162	4 (2·8)	1 (2·6)	1·000
Use of insect repellents	71 (38·2)	39 (30·0)	32 (57·1)	0·001*	56 (38·6)	14 (36·8)	1·000
Other	8 (4·3)	7 (5·4)	1 (1·8)	0·439	6 (4·1)	1 (2·6)	1·000
HL scores; Mean (SD)^a^							
Communicative HL score	10·5 (2·3)	10·4 (2·6)	10·6 (1·6)	<0·449	10·5 (2·3)	6·4 (1·5)	0·689
Critical HL score	6·5 (1·5)	6·4 (1·5)	6·6 (1·5)	0·327	10·3 (2·5)	6·6 (1·7)	0·569
Total HL score	16·9 (3·5)	16·8 (3·8)	17·3 (2·5)	0·337	16·9 (3·4)	16·9 (3·9)	0·950

Data are presented as numbers and proportions (%) unless otherwise specified.

Missing values were excluded, and the proportions were calculated using the total number of responses for the respective section as the denominator.

TBDs–Tick-borne diseases; JSF–Japanese spotted fever; LB–Lyme borelliosis; SFTS—Severe fever with thrombocytopenia syndrome; HL–Health literacy

*Significant differences between groups at *p* <0·01.

^#^Multiple answers were allowed.

^a^ Student t-test

^b^ Chi-square test

### 3.2. Attitudes and practices

The attitudes among the respondents towards TBDs are summarized in Table 3. More than half of the respondents (65·1%) were worried about being bitten by ticks, while women were significantly worried compared to men (*p* = 0·018). One hundred seventeen (62·9%) respondents were interested in preventing TBDs, particularly women (F—81·8%, M—57·1%, *p* = 0·001). With regard to practices, about four third of the respondents (n = 141) responded “yes” to taking preventive measures against tick bites with no significant difference in sex and age categories. Limiting skin exposure was the frequently used countermeasure by 130 (69·9%) respondents. In contrast, none of the respondents used light clothes. Fifty-eight (31·2%) of respondents reported that they use insect repellents. The practice of both countermeasures, limiting skin exposure (*p* = 0·023) and use of insect repellents (*p* = 0·001), were significantly higher among women (Table 3).

**Table 3:** Responses to attitudes and practices by sex and age.

	Total	By sex			By age		
		Male	Female	*p*	<65 years	≥65 years	*p* ^*a*^
**Attitudes**							
Worried about being bitten by ticks							
Yes	121 (65·1)	77 (59·7)	44 (78·6)	0·018*	94 (64·8)	26 (70·3)	0·567
No	64 (34·4)	52 (40·3)	12 (21·4)		51 (35·2)	11 (23·7)	
Interested in taking countermeasures							
Yes	117 (62·9)	72 (57·1)	45 (81·8)	0·001*	90 (63·4)	27 (75·0)	0·239
No	64 (34·4)	54 (42·9)	10 (18·2)		52 (36·5)	9 (25·0)	
**Practices**							
The practice of countermeasures against ticks							
Yes	141 (75·8)	94 (72·3)	47 (83·9)	0·097	108 (74·5)	31 (81·6)	0·403
No	45 (24·2)	36 (27·7)	9 (16·1)		37 (25·5)	7 (18·4)	
Countermeasures taken ^#^							
Limiting skin exposure	130 (69·9)	84 (64·6)	46 (82·1)	0·023*	100 (69·0)	28 (73·7)	0·692
Wear light-colored clothes	0 (0·0)	0 (0·0)	0 (0·0)		0 (0·0)	0 (0·0)	
Use of insect repellents	58 (31·2)	32 (24·6)	26 (46·4)	0·005*	45 (31·0)	12 (31·6)	1·000
Other	5 (2·7)	4 (3·1)	1 (1·8)	1·000	4 (2·8)	1 (2·6)	1·000

Data are presented as numbers and proportions (%).

Missing values were excluded, and the proportions were calculated using the total number of responses for the respective section as to the denominator.

*Significant differences between groups at *p* <0·05.

^#^Multiple answers were allowed.

^a^ *p*-value by chi-square test

### 3.3. Relationship between the practice of countermeasures against tick bites and study variables

The variations in each variable between the respondents who answered “yes” and “no” to the query “are you taking precautions against tick bites?” were analyzed, and the results are summarized in Table 4. The mean age of those recorded to take countermeasures was significantly higher (52·5 vs. 46·7 years old, *p* = 0·019). Majority of those who took precautions used newspapers (*p* = 0·006), TV (*p* = 0·002), internet (*p* = 0·002), and official publications (*p* <0·001) as their source of information. About knowledge, the respondents who were taking precautions were aware of LB (*p* = 0·024), SFTS (*p* <0·001), and preventive measures, including limiting skin exposure (*p* <0·001) and use of insect repellents (*p* <0·001) compared to those who are not taking precautions. Knowledge score was significantly higher among those who take precautions than those who do not (2·9 vs. 2·4, *p* = 0·031). The attitudes had a significant effect on the practice of countermeasures as the majority of those who were worried about being bitten by ticks (*p* <0·001) and interested in taking preventive measures (*p* <0·001) were taking precautions against tick bites. In addition, literacy score was significantly higher among the respondents who take precautions (17·3 vs. 15·8, *p* = 0·029).

**Table 4:** Relationship of study variables with the practice of countermeasures.

	Total	The practice of countermeasures against TBDs		*p*-value
		Yes	No	
Age; mean (SD)	51·1 (14·3)	52·5 (13·8)	46·7 (15·1)	0·019^a^*
Sex				
Male	130 (69·9)	94 (66·6)	36 (80·0)	0·097 ^b^
Female	56 (30·1)	47 (33·3)	9 (20·0)	
Type of household				
Living alone	15 (8·2)	10 (7·1)	5 (11·6)	0·037 ^b^*
Nuclear family	103 (56·3)	86 (61·4)	17 (39·5)	
Three-generation	62 (33·9)	43 (30·7)	19 (44·2)	
Other	3 (1·6)	1 (0·7)	2 (4·7)	
Experience of being bitten by ticks				
Yes	9 (6·8)	9 (8·7)	0 (0·0)	0·203 ^c^
No	123 (93·2)	95 (91·3)	28 (100·0)	
Sources of Information^#^				
Newspaper	50 (26·9)	45 (47·4)	5 (38·5)	0·006*
Magazines	20 (10·8)	17 (17·9)	3 (23·1)	0·413
Books	18 (9·7)	16 (16·8)	2 (15·4)	0·249
TV	63 (34·4)	56 (60·0)	7 (53·8)	0·002*
Radio	30 (16·1)	27 (28·4)	3 (23·1)	0·061
Internet	80 (43·0)	70 (73·7)	10 (76·9)	0·002*
Official publications	33 (17·7)	33 (34·7)	0 (0·0)	<0·001*
Knowledge of ticks score; mean (SD)	2·8 (1·3)	2·9 (1·2)	2·4 (1·5)	0·031^a^*
Knowledge of TBDs ^#^				
Scrub typhus	44 (23·7)	37 (27·2)	7 (16·7)	0·163
JSF	24 (12·9)	22 (16·2)	2 (4·8)	0·071
LB	14 (7·5)	14 (10·3)	0 (0·0)	0·024*
Tularemia	14 (7·5)	13 (9·6)	1 (2·4)	0·193
SFTS	44 (23·7)	42 (30·9)	2 (4·8)	<0·001*
Knowledge of preventive measures^#^				
Limiting skin exposure	154 (82·8)	133 (94·3)	21 (46·7)	<0·001*^b^
Wear light-colored clothes	5 (2·7)	4 (2·8)	1 (2·2)	1·000^c^
Use of insect repellents	70 (37·6)	67 (47·5)	3 (6·7)	<0·001*^b^
Other	8 (4·3)	3 (2·1)	5 (11·1)	0·021*^c^
HL score; Mean (SD)^b^	16·9 (3·5)	17·3 (3·1)	15·8 (4·2)	0·029 ^a^*
Worried about being bitten by ticks				
Yes	121 (65·4)	104 (74·3)	17 (37·8)	<0·001 ^b^*
No	64 (34·6)	36 (25·7)	28 (62·2)	
Interested in taking countermeasures				
Yes	117 (64·6)	99 (72·3)	18 (40·9)	<0·001 ^b^*
No	64 (35·4)	38 (27·7)	26 (59·1)	

Data are presented as numbers and proportions (%) unless otherwise specified.

*Significant differences between groups at *p* <0·05.

TBDs–Tick-borne diseases; JSF–Japanese spotted fever; LB–Lyme borelliosis; SFTS—Severe fever with thrombocytopenia syndrome; HL–Health literacy

^#^Multiple answers were allowed.

^b^ Chi-square test

^a^ Student t-test

^c^ Fishers exact test

### 3.4. Factors associated with the practice of preventive measures against tick-borne diseases

Multiple logistic regression analysis was performed to assess the factors associated with taking precautions for TBDs. Among the variables assessed, only age (*p* = 0·049), worried about being bitten by ticks (*p* = 0·046), and knowledge score (*p* = 0·029) had significant associations with practicing countermeasures. Increased age (OR = 1·036, 95% CI = 1·000–1·073), high knowledge score (OR = 1·462, 95% CI = 1·039–2·056), and worried about being bitten by ticks (OR = 0·396, 95% CI = 0·160–0·984) were significantly linked to the likelihood of practicing countermeasures against ticks. Interestingly, being interested in taking countermeasures did not have a significant association with the likelihood of practicing countermeasures (OR = 0.835, 95% CI = 0·310–2.252, p = 0.722).

## 4. Discussion

This study examined the KAP of people living in Niigata prefecture, Japan, regarding TBDs. To the best of our knowledge, this is the first study to assess KAP regarding TBDs among the general public in Japan. The knowledge regarding TBDs in the current study indicated that approximately 20% of the respondents knew about scrub typhus and SFTS, whereas less than or about 10% knew about other TBDs (JSF, TB, and Tularemia). Though not directly comparable, a study conducted in Scandinavia reported a low level of knowledge about ticks, LB, and TBE, especially among male and young age groups [18]. There are many grasslands that are flooded and have become wetlands in Niigata prefecture. Therefore, Niigata prefecture had been an area inhabited by Tsutsugamushi and several kinds of ticks [4, 19]. From the 1870s to the 1920s, deaths caused by TBDs were reported, with approximately 200 people being affected yearly and an approximate 30% mortality rate. Further, shrines for Tsutsugamushi (shrines to worship ticks) were built in Niigata prefecture to conduct prayers to eradicate scrub typhus, which suggests that scrub typhus might have been a familiar disease among the public in the past [20]. However, there have been only 0 to 4 deaths per year caused by scrub typhus in Japan recently [21]. The advent of diagnostic techniques and the development of efficient antibiotics are probably the key to decreased mortality rates associated with TBDs [2]. On the other hand, the awareness of scrub typhus among the public in Niigata prefecture might have been reduced, as observed in this study (Table 2), by the low occurrence of the disease.

Tick-borne SFTS was first confirmed in Japan in January 2013, and subsequently, a genetic diagnostic testing system for SFTS was established nationwide [3]. In recent years, cases of detecting the SFTS virus in the blood and feces of household cats and dogs, and cases of people with a history of bites from unwell cats developing SFTS and dying, have been reported. These incidences might be the reason for the awareness of SFTS among this study’s respondents (Table 2). A recent tick survey conducted in Niigata prefecture found 12 species in the area: *Dermacentor taiwanensis*, *H*. *flava*, *Haemaphysalis hystricis*, *Haemaphysalis japonica*, *H*. *longicornis*, *Haemaphysalis megaspinosa*, *I*. *ovatus*, *Ixodes nipponensis*, *Ixodes persulcatus*, *Ixodes monospinosus*, *Ixodes columnae*, and *Ixodes turdus* [4, 12], and this was a substantial change from the endemic species described in a previous survey in 1959 [13]. The most common tick species found in the prefecture were *H*. *flava*, *H*. *longicornis*, and *I*. *ovatus*, and these three species harbors *Rickettsia japonica*, which is the causative agent of JSF. The SFTS virus that causes SFTS is mainly transmitted by *H*. *longicornis*. *I*. *persulcatus* is a susceptible tick host for the pathogenic spirochetes that cause LB [12]. In addition, three causative species of human spotted fever; *R*. *asiatica*, *R*. *helvetica*, and *R*. *monacensis*, were found in *Ixodes* spp. and followed by *Haemaphysalis* spp. ticks [4]. With the available evidence of Niigata prefecture as an emerging risk area for TBDs in Japan, the low level of knowledge regarding TBDs and preventive measures (Table 2) found in this study is fundamental in initiating measures to improve public awareness.

About 80% of the study participants responded that they knew about reducing skin exposure, and 40% knew about using insect repellents as measures to prevent tick bites. Interestingly, the previous study that surveyed self-defense forces officers in Hokkaido, Japan, indicated nearly identical results on practicing countermeasures; “reducing skin exposure” 69·9% and “using insect repellent” 31·2% [15]. Moreover, a survey conducted in a high-risk endemic region in Finland reported that the rate of awareness on the limiting skin exposure and use of insect repellents were 81% and 21%, respectively [22]. These findings suggest that the overall knowledge of repellents is lower than that of limiting skin exposure, pointing out the need for raising awareness about repellents. Even though conflicting evidence exists on the effectiveness of repellents against ticks [23–25], their use has been recommended by many responsible institutions such as the Centers for disease control and prevention (CDC) in the United States, the European Center for disease prevention and control (ECDC), and the National Institute of infectious diseases in Japan. Further, a significant proportion of those aware of preventive measures in the current study: 85% of those who knew about limiting skin exposure and 94% of those who knew about using insect repellents responded that they are practicing the preventive measures against tick bites. These results emphasize the significance of improving the general population’s knowledge regarding preventive measures, which would enhance their practices.

The findings of this study indicated that the respondents’ attitudes toward TBDs had a significant impact on practices as respondents that worried about being bitten by ticks and interested in taking preventive measures had more than 70% rate of practicing countermeasures against tick bites. However, the rate of people practicing protective measures against tick bites is comparatively low as the difference between knowledge and actual practice was approximately 20% for both reducing skin exposure and using insect repellents. This suggests that many people do not take preventive measures even if they know, which was notably higher among men than women. Similarly, a recent survey conducted among German forestry trainees reported gaps in knowledge and adherence to practices. Only 8% of the forestry trainees used insect repellents, despite 32% believing that the repellents are effective, and only 17% wore long sleeves and pants as a protective measure [26].

In the current study, worried about being bitten by ticks and knowledge of ticks were identified as the factors significantly associated with the practice of countermeasures against tick bites (Table 5). Therefore, it is crucial to highlight the risks of TBDs and disseminate information on specific preventive methods for people who do not feel the need to take appropriate preventive measures against ticks and do not have sufficient knowledge about the issue. Five percent of respondents reported having been bitten by ticks in the past. In Japan, the fatality rate of tick-borne SFTS is 30%, indicating that TBDs are possibly life-threatening [27]. According to the health belief model, the ease of practicing a behavior is determined by balancing the perception of vulnerabilities and seriousness (susceptibility to illness and serious consequences) and the benefit of acting [28]. Therefore, by informing people about the risk and seriousness of TBDs, they will recognize the importance of taking preventive actions and, consequently, promote countermeasures against ticks. The primary sources of knowledge about TBDs were the internet and television, and the rate of people who preferred public institutions’ publications as a source of information was low. Therefore, it is necessary to consider how to disseminate correct knowledge to more people. Even now, local governments inform people about the risk of TBDs and specific preventive methods via the Internet. However, people with low interest cannot get such information because they do not access it unless they intentionally search. In the future, it is essential to consider methods of providing information effectively, for example, by selectively distributing information to people living in high-risk areas for TBDs, including agricultural areas, by utilizing targeting advertising methods. As the knowledge about TBDs was comparatively low among men, they may be important to target during information provision on TBDs. However, caution must be taken to induce precautionary behaviors towards TBDs without causing alarm to limit outdoor activities [18].

**Table 5:** Multiple logistic regression analysis with the practice of countermeasures against ticks as the dependent variable.

Variables	B	OR	95% CI	*p*
Sex (1: Males/ 0: Females)	-0·379	0·685	0·233–2·010	
Age	0·035	1·036	1·000–1·073	0·049*
Type of household				
Living alone	reference			
Nuclear family	0·891	2·437	0·096–61·814	0·589
Three-generation	1·765	5·844	0·352–97·057	0·218
Others	1·175	3·238	0·192–54·471	0·415
Sources of information (1: Yes/ 0: No)				
News paper	0·283	1·327	0·274–6·436	0·725
Magazines	0·159	1·172	0·229–5·994	0·849
Books	0·054	1·056	0·181–6·163	0·952
TV	-0·737	0·479	0·120–1·916	0·298
Radio	-1·649	0·192	0·037–1·001	0·050
Internet	-0·162	0·850	0·205–3·519	0·823
Official publications	-19·733	0·000	0·000–0·000	0·998
Knowledge of ticks score	0·380	1·462	1·039–2·056	0·029*
HL score	0·070	1·073	0·944–1·219	0·280
Worried about being bitten by ticks (1: Yes/ 0: No)	-0·926	0·396	0·160–0·984	0·046*
Interested in taking countermeasures (1: Yes/ 0: No)	-0·180	0·835	0·310–2·252	0·722
Hosmer-Lemeshow test	χ^2^ = 5·313 p = 0·724			

B—Regression coefficient; OR–Odds ratio; CI–Confidence interval of OR; HL–Health literacy

*p <0·05.

This study was conducted at a time when evidence on the TBDs in Niigata prefecture came to light. Therefore, this research is relevant and timely. This was the first study to assess KAP regarding TBDs among the general public in Japan. Thus, this study provides baseline information for future studies and initiating public health interventions to promote awareness. Nevertheless, there were some limitations of this study. First, we found missing responses in many questionnaires, and therefore the calculated proportions may not accurately represent the whole study sample. However, we have calculated proportions excluding missing responses for each item to get a clear idea when interpreting the results. Second, we calculated knowledge of ticks score based on answers to a set of questions, which is somewhat arbitrary and may not incorporate the weight of each question into the score. However, we believe that the score provides a fairly plausible estimate for the knowledge of ticks. Third, comparison of these findings is challenging due to differences in the questionnaires used in each study and a lack of a standardized questionnaire.

## 5. Conclusion

In conclusion, we identified gaps in knowledge and practices toward TBDs among people in Niigata prefecture, Japan. Knowledge of ticks and being worried about tick bites were the independent factors associated with taking preventive measures against tick bites. Therefore, it is crucial to disseminate information on TBDs and preventive measures and highlight the risks through communication tools. Further, epidemiological studies are recommended to assess KAP regarding TBDs in other high-risk groups and high-risk areas in Japan as limited data is available to date.
